# The estimation of environmental and genetic parental influences

**DOI:** 10.1017/S0954579422000761

**Published:** 2022-12-16

**Authors:** Jared V. Balbona, Yongkang Kim, Matthew C. Keller

**Affiliations:** 1 Institute for Behavioral Genetics, University of Colorado Boulder, Boulder, CO 80303, USA; 2 Department of Psychology & Neuroscience, University of Colorado at Boulder, Boulder, CO 80303, USA

**Keywords:** genetic nurture, heritability, nature and nurture, parental effects, vertical transmission

## Abstract

Parents share half of their genes with their children, but they also share background social factors and actively help shape their child’s environment – making it difficult to disentangle genetic and environmental causes of parent-offspring similarity. While adoption and extended twin family designs have been extremely useful for distinguishing genetic and nongenetic parental influences, these designs entail stringent assumptions about phenotypic similarity between relatives and require samples that are difficult to collect and therefore are typically small and not publicly shared. Here, we describe these traditional designs, as well as modern approaches that use large, publicly available genome-wide data sets to estimate parental effects. We focus in particular on an approach we recently developed, structural equation modeling (SEM)-polygenic score (PGS), that instantiates the logic of modern PGS-based methods within the flexible SEM framework used in traditional designs. Genetically informative designs such as SEM-PGS rely on different and, in some cases, less rigid assumptions than traditional approaches; thus, they allow researchers to capitalize on new data sources and answer questions that could not previously be investigated. We believe that SEM-PGS and similar approaches can lead to improved insight into how nature and nurture combine to create the incredible diversity underlying human behavior.

There is a substantial body of evidence showing that one of the most potent predictors of psychopathology is having a parent who experiences psychiatric illness themselves (Leverton, 2003; Stein et al., 2014). Nonetheless, while offspring resemble their parents on a wide variety of traits – from psychiatric illness to physical and social outcomes – the underlying causes of parent–offspring similarity have proven difficult for scientists to disentangle. Parents share half of their genetic effects with their children, leading to increased similarity for heritable traits. However, parents also tend to share relevant environmental influences with their children and will typically play an active role in shaping their children’s rearing environment – factors that may have enduring influences on certain offspring traits. Despite fueling scientific debate for over a century, this discrepancy between nature and nurture has seen little resolution and thus continues to be of interest for researchers and laypeople alike.

For much of the early 20th century, the notion of hereditarianism occupied a highly prominent role in the scientific community, largely as a result of the publication of Charles Darwin’s On the Origin of Species and the recognition of Gregor Mendel’s work on inheritance (Cravens, 1978). As a result, individual differences in psychiatric illness, cognitive ability, criminality, and alcoholism, as well as perceived racial and sex differences, were primarily attributed to genetic causes and thus believed to be beyond environmental intervention – a viewpoint that, for decades to come, had a massive influence on the field of psychiatry and on policy decisions around the globe (Cooper, 2001; Honeycutt, 2019). However, this prominence began to fade over time as the proceeding decades saw a (perhaps reactionary) paradigm shift, particularly within the nascent behavioral sciences. Specifically, the reigning hypotheses regarding behavior began to tilt heavily toward nurturing explanations, such that parenting practices were viewed as central in shaping nearly all aspects of an individual’s behavioral and cognitive traits. As a perhaps unintended consequence of this paradigm, parents (especially mothers) were blamed for their offspring’s developmental difficulties, atypical behaviors, and psychiatric illnesses. Maternal coldness, for example, was seen as the salient cause of later autism and schizophrenia in their offspring (Fromm-Reichmann, 1948; Kanner, 1949). These views began to be jettisoned in the 1970s as strong evidence for genetic and other biological influences on traits became difficult to ignore. In particular, twin studies – which allowed researchers to systematically quantify the roles of nature and nurture for individual human traits – seemed to consistently arrive at the same conclusion: evidence of substantial heritability was the norm across outcomes, whereas evidence of shared environmental effects (of which parental influences should be a part) was scant for many of the most studied traits, including cognitive ability, personality, and various neuropsychiatric disorders. Based on these findings, some scientists have again returned to the hereditarianist viewpoint that DNA is the primary driver in shaping who we are (Plomin, 2019).

However, despite its influence and continued use in examining nature and nurture, the classical twin design (*see* Table 1) is a poor tool for examining parental influences. The estimates of shared environmental influences from twin studies, which parental influences should contribute to, are often biased – downwardly in the presence of nonadditive genetic variance (e.g., interactions between variants) and upwardly in the presence of nonrandom mating (Keller & Coventry, 2005; Cloninger et al., 1979; Dalmaijer, in prep). Furthermore, estimates of shared environmental effects from twin studies only capture the aspects of parenting that induce similarity across offspring, which may not be a major contributor to the totality of parental influences for certain traits (Turkheimer & Waldron, 2000). Similarly, studies of adult twins will underestimate parental influences that fade over time, which could still be important to an individual’s developmental trajectory. Finally, twin studies require the assumption that the genetic and environmental influences on a trait are independent of one another, complicating the interpretation of results when this assumption is unmet.

This final assumption, regarding the independence of genetic and environmental factors, has proven particularly challenging in the context of understanding parental influences. When a heritable parental trait is having a direct influence on an offspring trait via the offspring’s rearing environment (a phenomenon termed *vertical transmission; see* Table 2), genetic and environmental factors are predicted to become correlated. This is because, in cases of vertical transmission, parents who exhibit a given heritable trait (e.g., antisocial behavior) not only provide to their children the genes that predispose to this trait but also a rearing environment that predisposes to it as well (e.g., one characterized by volatility, aggression, and callousness; Hart et al., 2021). This co-inheritance of genetic and environmental causal factors leads to a correlation between genetic and environmental influences, traditionally referred to as a *passive gene–environment correlation* in the behavioral genetics literature and more recently termed *genetic nurture* (*see* Figure 1). Although most genetically informed designs are unable to detect it, genetic nurture is a lurking presence that can bias genetic and environmental effect size estimates if unaccounted for. In studies of measured genetic polymorphisms, for example, effect size estimates will capture both the genetic effects of the polymorphism as well as any correlated effects of the rearing environment, leading to upwardly biased estimates for every truly associated polymorphism across the genome. Thus, it is important that genetic influences, vertical transmission, and genetic nurture be accounted for in order to tease apart the various factors that impact complex trait variation.

Fortunately, several designs have been developed that can disambiguate genetic effects, genetic nurture, and vertical transmission, producing estimates that are likely to be less biased in the presence of these factors. Here, we provide a brief nontechnical overview of traditional and recent approaches for examining the sources of parent–offspring similarity, with a particular focus on our own approach, structural equation model (SEM)-polygenic score (PGS). As described below, SEM-PGS builds upon recent insights into how to use parent and offspring PGSs to elucidate the cause of parent–offspring similarity, but does so using SEMs and other principals developed in extended twin family designs in an effort to derive estimates that are less biased than previous approaches (Balbona et al., 2021).

## Traditional approaches to understanding parental influences

### Extended twin family designs

Extended twin family designs (*see* Table 1) are extensions of the classical twin design that disambiguate the sources of similarity between close relatives, including parent and offspring, by modeling data from twin pairs and the twins’ family members. While the different types of extended twin family designs vary in their specifics, they all utilize parents and offspring, along with the various genetic relationships induced by including both identical and fraternal twins in the data. For example, the Children of Twins design is a type of extended twin family design that models data from pairs of adult twins and their offspring. The logic behind this approach is that the children of *identical* twins are as genetically related to their parent (who may influence a given trait via vertical transmission) as they are to their aunt/uncle (who does not influence the trait via vertical transmission), while the children of *fraternal* twins share the usual degree of genetic relationships; thus, part of the evidence for vertical transmission comes from higher parent–offspring than avuncular covariance among identical twins. Many other such unique relationships exist in extended twin family designs that, when fit simultaneously, allow researchers to derive potentially unbiased estimates of the variation due to several sources of relative similarity, including vertical transmission, genetic nurture, and additive genetic effects (Keller et al., 2010).

**Figure 2.**
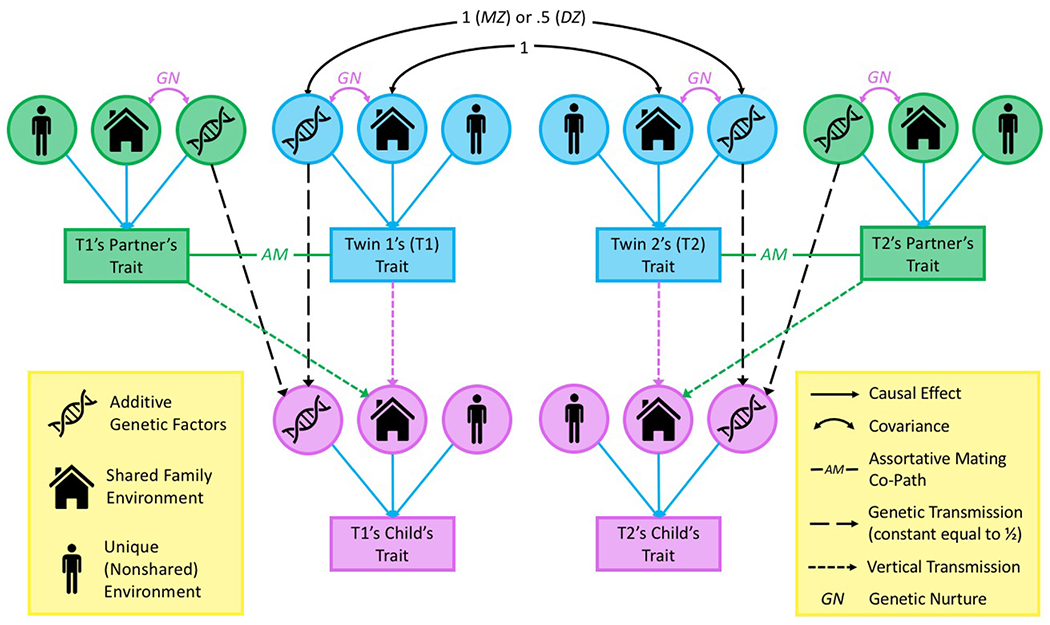
An illustration of how extended twin family designs build upon the classical twin design framework. The classical twin design (shown above in blue) compares covariances between identical/monozygotic (MZ) and fraternal/dizygotic (DZ) twins’ traits in order to estimate three sources of trait variation: (1) additive genetic factors, which are shared completely by identical twins and 50% by fraternal twins; (2) common/shared environmental factors, which are completely shared by both identical and fraternal twin pairs; and (3) unique/nonshared environmental factors, which are definitionally unique to each individual. By adding in twins’ offspring (shown in pink), the model becomes a Children of Twins design, in which vertical transmission and genetic nurture are estimable. Finally, incorporating the twins’ partners (shown in green), provides additional information on vertical transmission/genetic nurture and allows for the effects of assortative mating to be fully accounted for, avoiding a potentially large source of bias; in lieu of the twins’ partners this information can also be obtained by modeling data from the twins’ parents.

Altering the types of relatives included in these models allows researchers to address different potential causes of trait variation, making extended twin family designs adaptable to the data and question of interest (see Figure 2). Moreover, by modeling data from either the parents or spouses of twins, these models can account for the complicating influences of *assortative mating* – the tendency for mates to be more alike on various traits than expected by chance – which we discuss in greater detail below. Much of the flexibility and extensibility of extended twin family designs have been made possible by the use of SEM (Wright, 1934), which provides a number of advantages over other approaches (*reviewed in* Table 3). Nonetheless, several issues limit the utility of these designs. Foremost, the information used to estimate parameters in extended twin family design models comes solely from covariances between relatives’ phenotypes – genotypes are unmeasured and thus genetic (co)variances must be inferred from phenotypic (co)variances – which means that the accuracy of estimates depends strongly on a number of difficult-to-verify assumptions about the causes of phenotypic similarity between various relative types (Eaves, 1976; Keller et al., 2010). Related to this, extended twin family designs typically account for one particular type of assortative mating – equilibrium primary phenotypic assortative mating – which occurs when individuals are mating based on phenotypic similarity, and have done so for many generations. However, assortment may have only begun in the last generation or two, and it may be due to similarity in nonheritable background social factors (e.g., geographic location). Such alternative types of assortative mating are possible (if not typical) and can lead to serious biases when they occur (Keller et al., 2010). Third, extended twin family designs require samples that are difficult to collect and so are typically small and not publicly shared. Finally, the incorporation of additional relative classes improves statistical power and reduces bias, but it comes with the cost of immense added complexity and often requires larger sample sizes to achieve sufficient power (McAdams et al., 2014; Posthuma & Boomsma, 2000). These latter two limitations are barriers of entry to the widespread use of extended twin family designs and limit the number of findings that exist based on them.

### Adoption studies

Because of their power and simplicity, adoption studies have long been viewed as a gold standard for disambiguating the factors that lead to parent–offspring similarity (Horn et al., 1979). This design capitalizes on the assumption that, in cases of adoption, similarity between offspring and their biological parents is due to genetic (but not environmental) causes, whereas similarity between offspring and their adoptive parents is due to environmental (but not genetic) causes. Therefore, assuming that the child’s genotype is uncorrelated with that of their adoptive parents (i.e., that there was no selective placement) and that no vertical transmission occurred between the biological parent and their child prior to adoption, estimates of vertical transmission using adopted children should be free from genetic influences or bias due to genetic nurture (Jaffee et al., 2012; Rutter et al., 2001). Additionally, estimates of the magnitude of genetic effects can be obtained by examining the covariance between biological parents and their adopted-away offspring, or by comparing covariances within adoptive families to those within demographically matched biological families.

While powerful, adoption studies share many of the limitations of extended twin family designs: their estimates depend strongly on assumptions about the underlying causes of phenotypic covariance between relatives, they typically do not account for assortative mating, and they require samples that are difficult to collect and are therefore often small and proprietary. The assumption that biological parent-adopted away offspring similarity is due solely to genetic factors can be especially problematic given evidence that the prenatal environment (provided by the biological mother) plays a vital role in the development of many traits – especially those relevant to an individual’s physical and neuropsychiatric health (Salam et al., 2014). Additionally, the assumption that adoptees are placed randomly is often difficult to verify, given that parents and adopted offspring who are genetically uncorrelated for the trait of interest (e.g., educational attainment) may still be genetically correlated on a separate, potentially relevant variable (e.g., intelligence or a dimension of personality); violations of this assumption can upwardly bias estimations of vertical transmission (Shih et al., 2004). Finally, the generalizability of adoption studies can be limited by the restricted range of adoptive home environments and by the potential genetic and environmental dissimilarity of adoptees to the general public (Rhee & Waldman, 2002). Despite these limitations, adoption studies remain an important tool for understanding the causes of parent–offspring similarity.

## Recent approaches that use measured genetic data to understand parental influences

In addition to those listed in Table 3, one of the great strengths of SEMs is their ability to represent unobserved parameters as *latent variables* – variables that, despite not being measured themselves (or in some cases being impossible to measure at all), still serve as hypothesized sources of variation among the measured variables. In the case of extended twin family designs and adoption studies, latent variables are used to model the influence of genetic factors on trait variation without requiring the collection of any genotypic data; thus, SEM made the study of genetic and environmental effects possible for the decades prior to human genome sequencing.

Nonetheless, in recent years, researchers have found creative means through which measured genomic data, particularly *polygenic scores* (PGSs, also referred to as “*polygenic risk scores*”), can be used to estimate both genetic and nongenetic parental influences. An individual’s PGS serves as an indicator of their genetic predisposition toward a given trait and is calculated as the count of trait-increasing alleles present in their genome, weighted by each allele’s degree of association with the trait (*for a review, see* Wray et al., 2021). The weights used in calculating PGSs are based on results from *genome-wide association studies* (GWAS, pronounced “gee-wos”) – hypothesis-free observational studies that examine differences in allele frequencies between cases and controls, or between people with different values on a continuous trait. Once calculated, PGSs are first validated using cohorts with known case/control status, after which they can be used for prediction in independent samples. Thus, by condensing the effects of many genetic variants (typically millions) into a single summative value for each individual in a sample, PGSs are a highly valuable, user-friendly tool for examining the genetic underpinnings of complex traits; this is particularly true for the study of psychiatric disorders, nearly all of which have been found to result from massively complex and polygenic etiologies (Hyman, 2018).

While PGSs have great potential for the study, detection, and treatment of psychiatric disorders, there remain several important considerations with regard to their use. First, the predictive ability of PGSs is greatly attenuated when used in samples that differ ancestrally from that of the original GWAS. Given that a significant majority of GWAS to-date have been conducted in individuals of European descent, this unfortunately means that across most traits, PGS accuracy greatly suffers when applied in non-European samples – a problem that limits their applicability in clinical and research settings, and one that researchers are working to mitigate through the recruitment of larger and more diverse GWAS samples (Martin et al., 2019; Peterson et al., 2019). Additionally, across all ancestry groups, PGS explain a fraction of total trait variation (Eichler et al., 2010). The reasons for this are twofold: First, polygenic risk scores are only capturing one type of genetic contribution to risk (i.e., the additive effect of measured genetic variants), and genetic contributions are only one component of overall risk (Turley et al., 2021). Second, each variant’s effect size estimate contains a somewhat large degree of error relative to its true effect, and this error is ultimately summed across the genome when creating a PGS. Importantly, while the relative degree of this estimation error depends in part on the sample size used for the initial GWAS (with larger samples being associated with a decrease in noise and corresponding increase in the PGS’s predictive ability), the size of the sample in which the PGS is being applied has no impact on the predictive ability of the PGS. Nonetheless, even when derived from large samples, PGS predictive power is typically some fraction (e.g., 1%–50%) of the total trait heritability (Eichler et al., 2010), making PGSs an imperfect (though reliable) reflection of an individual’s genetic predisposition toward a given outcome.

Despite these limitations, a growing number of publications have used PGSs to elucidate the role of nongenetic parental effects. Here, we focus on the best-known approach, introduced by Kong et al. (2018) as a means to estimate the degree of genetic nurture underlying variation in educational attainment.

### Kong et al. (2018) and other recent approaches

**Figure 3.**
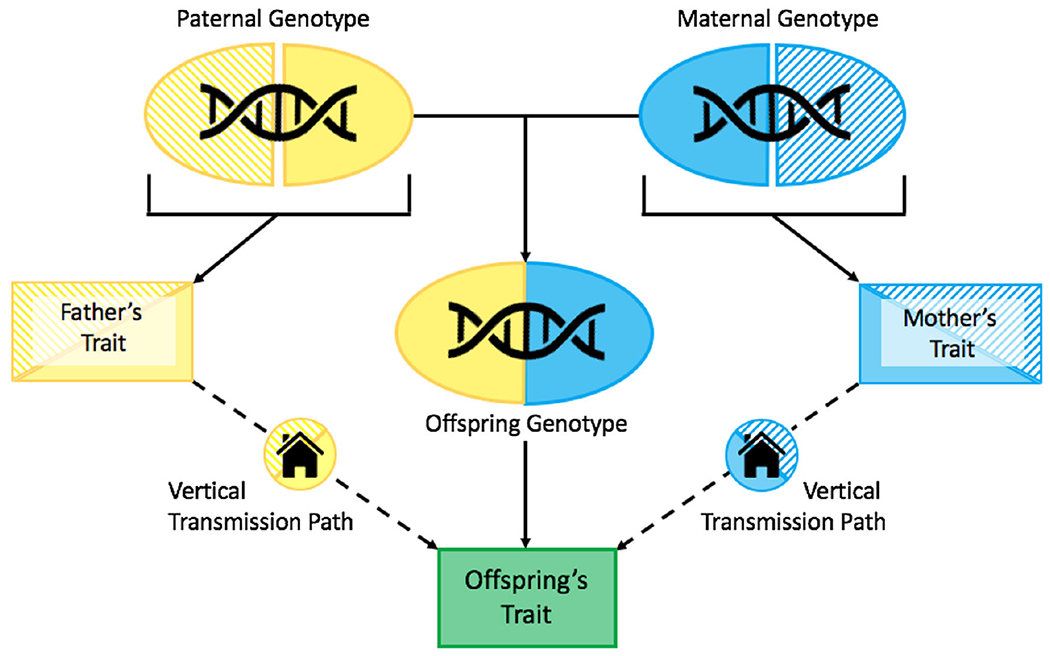
Schematic of the Kong et al. approach (adapted from Kong et al., 2018) For each parents-offspring trio in their sample, Kong et al. constructed four PGSs (each illustrated above as semi-ellipses): Two from the portion of the genome that parents transmitted to their child (depicted using solid colors) and two from the portion of the genome that parents did not transmit to their children (depicted as striped). Both the transmitted and nontransmitted PGS’s directly influence the parental traits, which in turn have an effect on the offspring’s phenotype via vertical transmission/genetic nurture. However, only the two transmitted PGSs – which together form the offspring’s full PGS – have a direct effect on the offspring trait that is not mediated by the familial environment. Thus, by comparing the transmitted and nontransmitted PGSs’ associations with the offspring’s phenotype, researchers can estimate the relative magnitudes of genetic nurture and direct genetic effects.

The Kong et al. study used genetic and phenotypic data from ~22K Icelandic trios (i.e., offspring, their mothers, and their fathers) to divide each parent’s genotype into two groups: the set of alleles that the parent *transmitted* to their child (which are therefore shared between the parent and offspring), and the set of alleles that the parent did *not transmit* to their child. The authors then created separate educational attainment PGSs from each of these four sets of alleles, such that there were two transmitted haplotypic PGSs (one from the father and one from the mother, together comprising the offspring’s full PGS) and two nontransmitted haplotypic PGSs for each family (see Figure 3).

Unlike the transmitted PGSs (denoted PGS_T_) the nontransmitted PGSs (PGS_NT_) are, by definition, genetically unrelated to the offspring. Therefore, assuming that any potential confounding influences (e.g., assortative mating and population stratification) have been properly controlled for, associations between PGS_NT_ and the offspring trait cannot be due to shared genetics. This association is instead most likely due to PGS_NT_’s influence on the parental trait, which in turn influences the offspring trait via vertical transmission. In this way, the Kong et al. approach shares logic with the adoption design. Specifically, PGS_NT_ serves a role analogous to that of an adoptive parent (who influences the offspring via nongenetic means), whereas PGS_T_ serves a role analogous to that of a typical biological parent (who influences the offspring via both genetic and nongenetic means). By comparing the association of PGS_T_ with the offspring phenotype to the association of PGS_NT_ with the offspring phenotype, direct genetic effects can be parsed from the genetic nurture effect.

**Figure 4.**
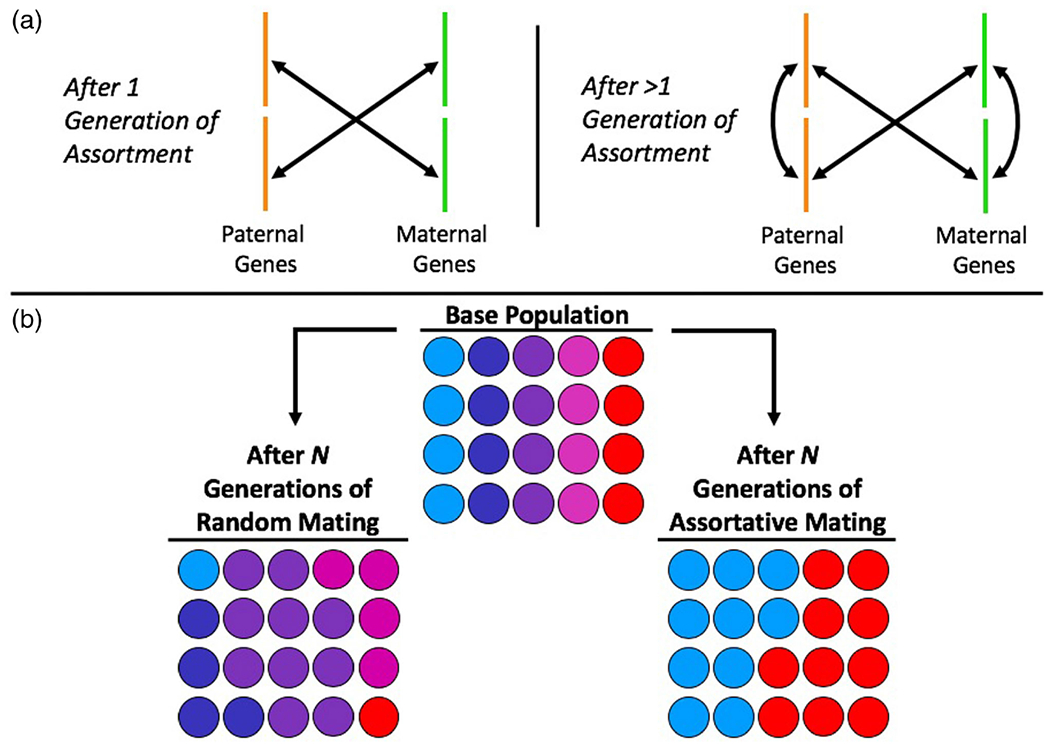
Assortative mating increases the phenotypic variance in a population and will lead to correlated genotypes within and between mates. (a) Adapted from Kong et al. (2018). For heritable traits, assortative mating implies that mates have correlated genotypes. Therefore, a single generation of assortment (i.e., in the offspring’s parental generation) will lead to covariances between parents’ genotypes, such that the genes inherited from one parent will covary with the genes inherited from the other parent. If assortment has occurred for more than one generation (i.e., in both the parental and grandparental generations and perhaps before), the genes inherited from one parent will also be correlated with the other genes inherited from that same parent. For example, the genes originally passed down from one’s maternal grandmother will be correlated with those from their maternal grandfather, both of which are later transmitted to the offspring by their mother. (b) For a random mating population, quantitative traits – in this example, hue from blue to red – will adopt a normal distribution over time, such that most people will fall somewhere in the middle of the trait’s distribution with few individuals on the extremes. Conversely, the variation in traits under assortative mating will increase because alleles of similar effects will tend to congregate in the same genomes. At the extreme, as illustrated here, trait distributions can become bimodal over time (although assortative mating this extreme is probably rare).

It is worth noting that the Kong et al. study was not the first to utilize transmitted and nontransmitted PGSs to examine parent–offspring similarity. To our knowledge, this insight was first proposed by Zhang et al. (2015) for Mendelian Randomization studies – an approach that uses genetic associations as instrumental variables in order to test causal hypotheses regarding the effects of modifiable risk factors on outcomes (for a review, see Davies et al. (2018)). Warrington et al. (2018) and Evans et al. (2019) later built upon this idea by incorporating PGSs of mothers and offspring into SEMs, thereby properly accounting for the recursive relationships that arise in this context, such as genetic effects upon which the PGS is based being overestimated due to genetic nurture. Nonetheless, the work by Kong et al. built upon these previous approaches in several important ways. Unlike other studies (which viewed genetic nurture as a nuisance variable to be controlled for), Kong et al. chose to focus on genetic nurture itself, thereby attempting to estimate its full effect and compare it to the direct genetic effect. Kong et al., also incorporated data from fathers as well as mothers to distinguish paternal versus maternal genetic nurture effects. Additionally, unlike past methods, Kong et al. attempted to control for the potentially confounding influences of assortative mating. Because assortment on heritable traits implies that mates have correlated genotypes, a single generation of assortment will result in the genes an individual received from one parent being correlated with the genes they received from the other parent; meanwhile, if assortment has gone on for more than one generation, the trait-associated genes *within* each parent’s genome will be correlated with one another, adding an additional layer of complexity (Figure 4a). Thus, if assortative mating is not accounted for in the Kong et al. approach, a portion of the genetic nurture estimate (i.e., the association between PGS_NT_ and the offspring’s phenotype) could actually be driven by direct genetic effects via correlations between PGS_NT_ and PGS_T_, resulting in potential serious bias in the estimates of direct genetic effects, genetic nurture, and vertical transmission (Kim et al., 2021). This bias may be further exacerbated by the increase in population phenotypic variance that assortment induces (Figure 4b), which – depending on the study design – may be misattributed to either genetic (in studies using genome-wide approaches) or environmental (in many studies using twin-based approaches) sources if assortment is unaccounted for.

Despite its important advances, the Kong et al. approach also has its limitations. First, its estimates of genetic nurture and direct genetic effects are only the portions of those effects captured by the PGS; they are therefore downwardly biased to the degree that the PGS fails to explain the full trait heritability, which (for the reasons discussed above) implies a substantial downward bias for all traits currently. Second, Kong et al. stopped at estimating genetic nurture and did not attempt to estimate the vertical transmission effect itself, despite having the necessary information to do so. Third, the authors found evidence suggesting that assortative mating on their trait of interest (education level) did not occur until the sample’s parental generation, and that this assortment was based on between-mate phenotypic similarity, rather than background environmental similarity. As a result, their approach only accounts for this one specific type of assortment and will be biased under alternative scenarios. Finally, much of the math presented by Kong et al. was derived from first principals which, while impressive, means that it largely only applies to the specific case for which it was derived. As a result, it cannot easily be extended to other situations (e.g., multiple generations of assortative mating) or data structures (e.g., inclusion of other relative types).

Since the publication of the Kong et al. (2018) study, approaches such as relatedness disequilibrium regression (Young et al., 2018) and trio-based genome-wide complex trait analysis (trio-GCTA; Eilertsen et al., 2021) have attempted to estimate parental influences using genomic similarity at genome-wide polymorphisms between all individuals in samples of trios. While relatedness disequilibrium regression and trio-GCTA are intended to estimate the full additive and genetic nurture influences without bias, they underestimate the full variance due to vertical transmission because they only capture the portion of the vertical transmission effect that is correlated with parental genotype (Young et al., 2018); for a trait with low heritability, this underestimation can be severe. Moreover, these approaches do not account for assortative mating, which will bias all the estimates in various ways. For example, it is estimated that the heritability of height reported in Young et al (2018) was 20% lower than its true value (Kemper et al., 2021) due to the influence of assortative mating.

## SEM-PGS models

**Figure 5.**
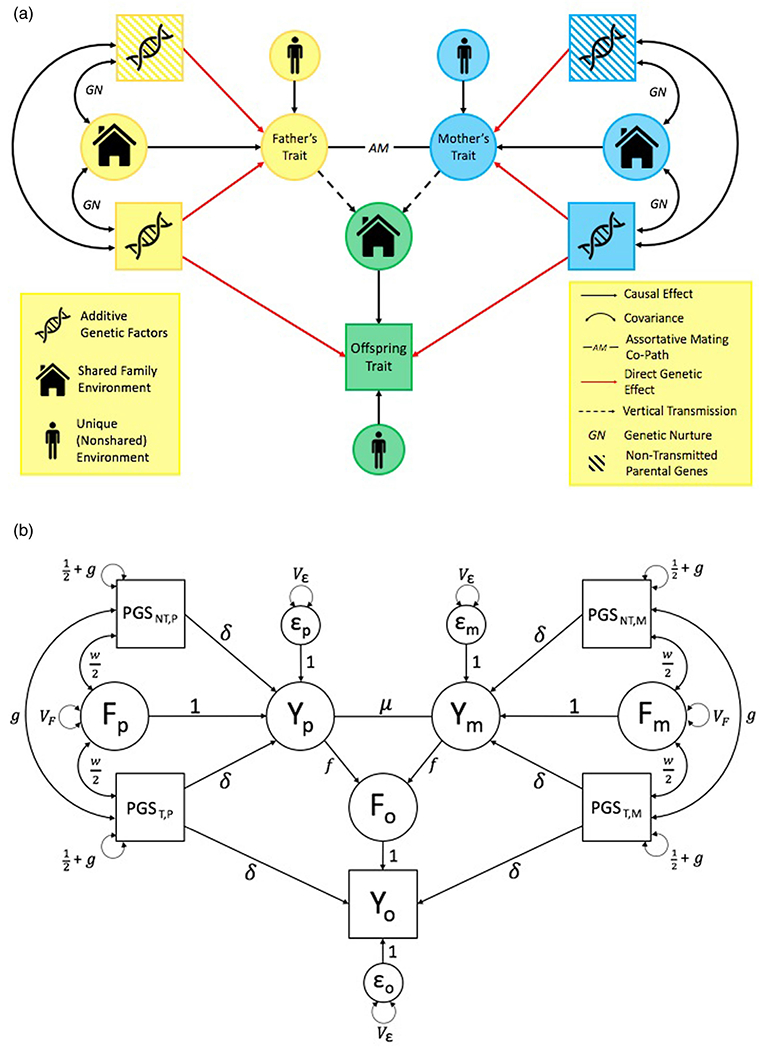
SEM-PGS utilizes many of the same constructs used in extended twin family designs and obtains its estimates via path tracing. (a) As shown, many of the elements of the SEM-PGS models are also common to extended twin family designs. As with extended twin family designs, SEM-PGS models each individual’s additive genetic effects, their familial environmental effects, and the covariance between the two (i.e., genetic nurture). Both approaches also model the effects of the individuals’ unique/nonshared environments and use data on partners (in this case, the offspring’s parents) to account for assortative mating. Of course, they differ from extended twin family designs in their utilization of measured genetic data – specifically their use of transmitted (shown as solid colors) and nontransmitted (shown as striped) PGSs as hypothesized sources of phenotypic variation. (b) SEMs can be depicted using path diagrams, such as the one shown above, in which hypothesized relationships between observed variables are shown. In path diagrams, single-headed arrows signify causal relationships from one variable to another, with their associated path coefficients (e.g., δ above) being akin to partial regression coefficients. Double-headed arrows, meanwhile, signify covariances between two variables, or variances when connecting a variable to itself. To determine expected (co)variances between two variables using a path diagram, one must identify all ‘legitimate’ paths – that is, paths which abide by a given set of rules (described in Balbona et al., 2021 and elsewhere) – that connect the two variables (for expected covariances) or that connect a variable to itself (for expected variances). For example, in examining the covariance between PGS_NT,p_ and Y_o_, one of the legitimate paths would be PGS_NT,P_ → Y_p_ → F_o_ → Y_o_, one of the genetic nurture paths. Another path would be PGS_NT,P_ → Y_p_ → Y_m_ → PGS_T,p_ → Y_o_, illustrating how assortative mating induces a correlation between nontransmitted alleles and the offspring trait via the transmitted alleles.

We recently developed a series of models for estimating parental effects that instantiate the underlying logic of Kong et al. (2018) into a series of SEMs that are based on principals developed in the extended twin family design literature (Figure 5a; Balbona et al., 2021). We believe that this instantiation is simple but consequential, turning Kong et al.’s insight for a specific analysis into a novel and extensible genetically informative design. Figure 5b shows a basic SEM-PGS model that illustrates many of the core ideas common to all the models. As shown, all SEM-PGS models use at least the following five observed pieces of information (depicted as squares in the diagram): the *nontransmitted* maternal and paternal haplotypic PGSs (PGS_NT,m_ and PGS_NT,p_, respectively), the *transmitted* maternal and paternal haplotypic PGSs (PGS_T,m_, and PGS_T,p_), and the offspring phenotype (Y_o_). Meanwhile, paternal, maternal, and offspring familial environments (F_p_, F_m_, and F_o_, respectively) are modeled as latent variables (depicted as circles). Note that the parental phenotypes (Y_p_ and Y_m_) are also operationalized as latent variables in this model. Thus, SEM-PGS does not require observed parental traits to estimate vertical transmission, although including them is useful for estimation of the full direct genetic and genetic nurture effects.

These observed variables create a 5-by-5 variance–covariance matrix from which seven parameters are estimable: (1) δ, the direct effect of a PGS on the individual’s phenotype; (2) *f*, the direct effect of a parental phenotype on an offspring phenotype (i.e., vertical transmission); (3) *g*, the increase in PGS (co)variances that results from assortative mating in the previous generation(s); (4) *w*, the covariance between PGSs and their rearing environment (i.e. genetic nurture); (5) *μ*, the assortative mating coefficient; (6) *V*_*F*_, the proportion of phenotypic variance due to vertical transmission; and (7) *V*_*ε*_, the residual phenotypic variance. All of these parameters can be estimated using model-fitting software (such as OpenMx; Boker et al., 2011), which attempts to mimic as closely as possible the observed variance–covariance matrix with the one implied by the maximum likelihood estimates of the model’s unknown parameters.

The use of SEM in SEM-PGS has several important advantages. First, SEM-PGS is designed to estimate *V*_*F*_ while controlling for genes shared between parents and offspring. Unlike trio-GCTA, relatedness disequilibrium regression, and the Kong approach, SEM-PGS estimates, the full *V*_*F*_ – that is, the total variation in an offspring trait due to vertical transmission from a parental trait – even when the PGSs being used have poor predictive ability. Second, SEMs provide a set of rules that simplify otherwise near-intractable recursive mathematical equations in models involving vertical transmission and assortative mating. The simplicity of SEM-PGS allows its models to be readily extended or adapted depending on the data. Third, although SEM-PGS was designed with trio data in mind, we have shown that by using full information maximum likelihood (Schafer & Graham, 2002), our estimates are unbiased by data missing at random (Kim et al., 2021). Therefore, SEM-PGS does not require trio data, but can instead be used on data containing relative pairs (including parent–offspring, spouse, and sibling pairs), such as what exists sporadically in large biobanks like the UK Biobank. Individual-level data can also be leveraged to boost statistical power. Fourth, as already noted, assortative mating is a potential confounder of estimates in all the designs discussed above. SEM-PGS models can detect and fully account for different types of assortative mating, including assortment that is at equilibrium (having occurred for many generations) or at disequilibrium (having begun relatively relatively) and assortment that is based on phenotypic, background environmental, or genetic similarity.

In Kim et al. (2021), we demonstrate that all of the specific SEM-PGS models we developed work as designed, producing unbiased estimates when their assumptions are met. Nevertheless, as with any model, SEM-PGS has important limitations and caveats that need to be considered in conducting analyses and interpreting results. First and most obviously, while SEM-PGS models require less stringent assumptions than previous approaches, their estimates can still be biased when assumptions are unmet or its model is misspecified. For example, current SEM-PGS models assume that the PGS is as predictive in offspring as it is in parents, which could be violated if gene-by-age interactions exist. While the flexibility of the SEM-PGS approach would allow for this type of situation to be modeled if it is detected, estimates from SEM-PGS would be biased otherwise; thus, rather than applying SEM-PGS to data “out of the box”, it is important to carefully vet its assumptions and make adjustments accordingly. Second, while SEM-PGS estimates of *V*_*F*_ (the proportion of phenotypic variance due to vertical transmission) should be unbiased regardless of the PGSs predictive ability, the standard errors for those estimates will increase as the PGSs predictive ability decreases (Kim et al., 2021). Therefore, for parental effects to be precisely estimated, PGSs need to be adequately predictive (e.g., *r*^2^ > ~.05 for a sample size of 8K trios or 16K parent–offspring pairs, or *r*^2^ > ~.02 for 30K trios or 60K parent–offspring pairs). A corollary of this limitation is that SEM-PGS can only examine parental traits for which relatively large external GWASs (and therefore adequately predictive PGSs) exist. While the number of traits analyzed in GWAS and their sample sizes are growing rapidly, many traits relevant to parenting remain unexamined in GWAS.

In addition to these limitations, an important caveat regarding the interpretation of SEM-PGS estimates should also be noted: estimates of *V*_*F*_ from SEM-PGS do not capture the variance of the *entire* influence of parents on a given offspring trait, but rather the impact that *the parental trait being captured by the PGSs* has on the offspring trait. For example, if one were to examine influences of parental depression on offspring depression using SEM-PGS, the estimates of *V*_*F*_ would capture the influence of parental depression – as well as any traits genetically related to parental depression – on offspring depression, while being blind to the role of other parental traits (e.g., externalizing disorders) to the degree that they are genetically uncorrelated with depression. We view this issue as both a strength and a limitation to the model – a strength because insight into the specificity of the influences of parental traits is itself important, and a limitation because the total influence of vertical transmission on a trait will be underestimated. Nonetheless, to partially address this issue, we are currently working on multivariate extensions of the current univariate SEM-PGS models that would allow for the estimation of a parental trait’s influence on a different offspring trait. While previous genetically informed studies have found evidence of cross-trait parental effects (de Zeeuw et al., 2020; Kong et al., 2018; Pingault et al., 2021; Torvik et al., 2020), these studies have not been conducted multivariately. Instead, they have inserted different parental (e.g., educational attainment) and offspring (e.g., health) traits into an otherwise univariate model – an approach that does not properly account for within-trait genetic nurture and vertical transmission, cross-trait genetic effects, or assortative mating. Thus, extending SEM-PGS models to be multivariate using standard techniques developed in the behavioral genetics literature (Vogler & Cockerham, 1985) will hopefully improve our understanding of cross-trait parent–offspring associations.

## Discussion

Given the enormous economic, social, and personal burden that psychiatric illness places on individuals and on society, examining the factors that contribute to its onset and trajectory is of immense societal importance (Eaton et al., 2008). This is particularly true psychiatric illness in children, which has been found to negatively affect healthy development and to increase the risk of later adulthood illness and dysfunction (Copeland et al., 2021). At present, about one in six US children aged 2–8 years has a diagnosed mental, behavioral, or developmental disorder (Cree, 2018), and this rate increases to nearly one in two by adolescence with depression and anxiety making up a majority of cases (Ghandour et al., 2019; Merikangas et al., 2009). Understanding the causes of childhood psychopathology – as well as the consequences that parental psychopathology has on the offspring’s development – is therefore crucial.

To this end, the last decade has seen an explosion of research on the topic of parental influences, and with good reason – recent studies that control for shared genetics between parents and offspring are providing mounting evidence for the direct roles that parental behavior has on offspring traits (Jami et al., 2021). While most of these studies have relied upon traditional (e.g., adoption and extended twin-family) approaches, a growing number of newer designs have repurposed measured genetic data to better elucidate the causes of parent–offspring similarity. It is in this vein that we recently developed SEM-PGS – an approach for estimating parental effects that builds upon both traditional SEM-based designs and newer approaches that utilize genome-wide data. Of course, SEM-PGS is one of many newer methods that takes advantage of the power and flexibility of SEM to model measured genotypic data (e.g., Grotzinger et al., 2019; Warrington et al., 2018). With the introduction of such approaches, the gap between measured genetic and traditional family-based approaches appears to be narrowing – part of an exciting evolution underway in the field of behavioral genetics and one that will allow enduring questions to be answered in new ways. While we celebrate this transition, we caution against the notion that approaches that use genomic data will replace traditional ones or that either approach is superior to the other. As both approaches carry their own strengths, limitations, and assumptions, the triangulation of results will provide a clearer understanding of the phenomena under study. Thus, the various approaches in behavioral genetics, both traditional and novel, are complementary, not competing (Friedman et al., 2021).

The study of parental effects using genetically informative designs is important for many reasons. Most obviously, a central goal of behavioral genetics (and the behavioral sciences broadly) is to accurately estimate the various causes of human behavioral variation, particularly with regard to traits that negatively impact an individual’s health and development. Having designs that can accurately capture how parents influence offspring and the strength and durability of such influences is crucial to this goal. Related to this, the presence of vertical transmission for a trait implies the existence of genetic nurture, which in turn implies that genetic influences for such traits are overestimated by many existing genome-wide approaches (such as GWAS). Understanding which traits are influenced by vertical transmission is therefore important for interpretation of GWAS results and methods that rely on GWAS data, such as genome-based restricted maximum likelihood (GREML, a widely used approach for estimating additive genetic variance; Yang et al., 2011). Furthermore, access to designs that accurately estimate parental influences can help correct erroneous prior conclusions regarding the negligible influence of vertical transmission for certain traits, while potentially also corroborating its seeming lack of importance for other traits. Having such designs at scientists’ disposal may also direct focus to traits that are more likely to be influenced by vertical transmission or on developmental periods where such influences are greater.

Finally, in addition to the above points, understanding that certain traits are, in part, influenced by parenting is important to know as it is potentially actionable information. Breaking the chain of transmission in a single individual can reverberate down the generations. Thus, to the degree that risk for psychiatric illness is inherited via vertical transmission, interventions aimed at improving the functioning of parents with psychiatric illness would also reduce the burden in their children and potentially their grandchildren.

## Figures and Tables

**Figure 1.**
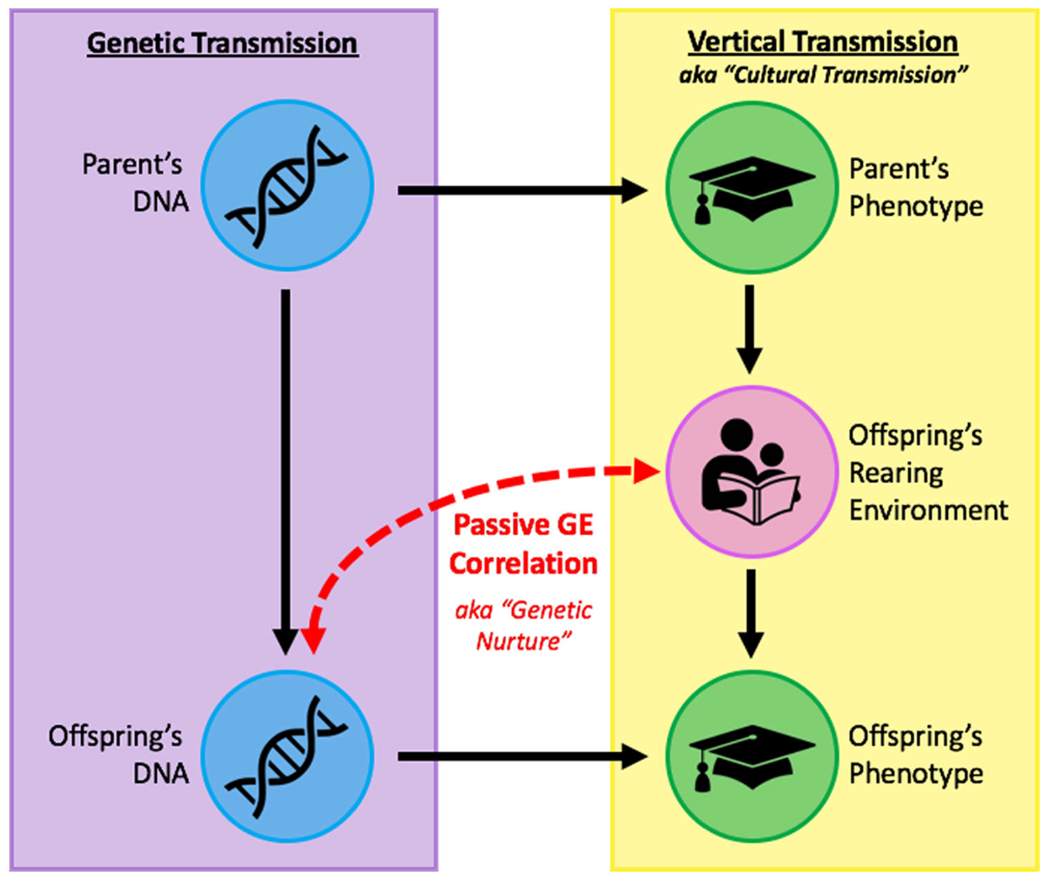
The co-occurrence of genetic transmission and vertical transmission will necessarily result in passive gene–environment covariance. In this example, highly educated parents provide to their child not only genes conducive to higher education but also a rearing environment that values and prioritizes education. This environment may include parental activities such as reading to the child, assisting with homework, or encouraging a positive attitude toward schooling. Thus, the offspring’s environment is influenced by the parental genes, causing the offspring’s genes and environment to be correlated with one another rather than independent – a phenomenon termed “passive GE correlation” (or more recently, “genetic nurture”) in the behavioral genetics literature.

**Table 1.** Summary of several genetically Informed approaches of estimating parental effects

	Requires	Utilizes:		Can estimate/account for:		
		Measured Genomic Data	Structural Equation Modeling	Vertical Transmission	Genetic Nurture	Assortative Mating
**Classical Twin Design**	Twin pairs	No	Yes	No	No	No

**Extended Twin Family Designs**	Twin pairs, and various combinations of the twins’ children, parents, and spouses	No	Yes	Yes	Yes	Only if AM is phenotypically driven and at equilibrium; biased otherwise

**Adoption Study**	Adoptees and their adoptive parents; biological parents can also be included	No	Yes	Yes	Yes	Only if AM is phenotypically driven and at equilibrium; biased otherwise

Kong et al. (2018)	Offspring and their parents (i.e., Trios)	Yes	No	Theoretically yes, but has yet to be used for this	Yes	Only if AM is phenotypically driven and has gone on for 1 generation; biased otherwise

**Relatedness Disequilibrium Regression**	Offspring and their parents (i.e., Trios)	Yes	No	Yes	Yes	No

**Trio-GCTA**	Offspring and their parents (i.e., Trios)	Yes	No	Yes	Yes	No

**SEM-PGS**	Offspring and their parents (i.e., Trios)	Yes	Yes	Yes	Yes	Yes

One of the most prominent discrepancies between the methods above lies in their respective abilities to estimate various types of assortative mating (AM). While AM can, and often is, driven by phenotypic similarity between mates, it may also be due to similarity on nonheritable environmental factors (such as one’s birthplace), or on genetic similarity. This distinction is important, as phenotypically (but not environmentally) driven AM will alter the genetic architecture of traits in a given population, biasing the estimates of trait heritability from a variety of study designs.

**Table 2.** Glossary of key genetics terms

Term	Definition
Heritability (*h^2^*)	The proportion of variance in a trait that is due to genetic variation between individuals as opposed to environmental variation. “Heritable” traits are thus those that have, at least to some extent, an underlying genetic basis
Additive genetic effects	The proportion of heritability contributed by the sum of multiple genetic variants’ individual effects. In contrast, nonadditive genetic effects include genetic dominance effects and epistasis (multiplicative interactions between two or more genetic variants). Additive genetic effects are what is typically being captured in estimates of heritability from GWAS, and from many twin-based studies.
Vertical transmission (VT)	The direct influence of a parental trait on an offspring trait, mediated by the offspring’s rearing environment (i.e., the home environment that parents help to create and shape)
Genetic nurture	Also referred to as *passive gene–environment correlation;* the covariance between an individual’s genetic effects and their rearing environment (both of which are generally provided by one’s biological parents)
Assortative mating (AM)	The tendency for people to preferentially mate with others who are similar to themselves. This preference may be conscious or unconscious, and the similarity may be with regard to the individuals’ phenotypes, genotypes, and/or environmental backgrounds
Polygenic Score (PGS)	Often referred to as a ‘polygenic risk score’; a score that reflects an individual’s relative genetic predisposition for a trait. PGSs are derived as the weighted sum of trait-increasing alleles within an individual’s genome, with the weights being derived from GWAS results
Genome-Wide Association Study (GWAS)	A hypothesis-free observational study that examines the extent to which each individual genetic variant in a given subset is associated with a trait; the specific variants used are typically single nucleotide polymorphisms (SNPs), chosen based on their minor allele frequency in the population

**Table 3.** Advantages of using structural equation models, taken in part from Keller et al. (2009) and Kline (2016)

Advantages of Structural Equation Modeling
1. SEMs can be easily adapted to the type of data at hand, what that data says about model assumptions, and the research questions of interest.
2. By finding estimates jointly rather than individually, the mathematical expectations of potential complex (e.g., recursive) relationships between variables can be greatly simplified. This can help reduce otherwise intractable math.
3. SEM directs focus to effect sizes and model evaluation rather than *p*-value thresholds.
4. SEMs require the user to carefully consider and describe the hypothesized causal processes underlying relationships in observed data.
5. Any model will be biased to the degree that its assumptions are unmet, but such assumptions are often implicit or ignored. SEMs encourage that model assumptions are made explicit and encourages testing of those assumptions.
6. Hypothesized causal processes and assumptions can be easily communicated via path diagrams – pictorial representations of causal models.

## References

[R1] Balbona JV, Kim Y, & Keller MC (2021). Estimation of parental effects using polygenic scores. Behavior Genetics. 10.1007/s10519-020-10032-w PMC8093180 33387133

[R2] Boker S, Neale M, Maes H, Wilde M, Spiegel M, Brick T, Spies J, Estabrook R, Kenny S, Bates T, Mehta P, & Fox J (2011). OpenMx: An open source extended structural equation modeling framework. Psychometrika, 76(2), 306–317. 10.1007/s11336-010-9200-6 23258944 PMC3525063

[R3] Cloninger CR, Rice J, & Reich T (1979). Multifactorial inheritance with cultural transmission and assortative mating. II. A general model of combined polygenic and cultural inheritance. American Journal of Human Genetics, 31(2), 176–198. 453202 PMC1685756

[R4] Cooper B (2001). Nature, nurture and mental disorder: Old concepts in the new millennium. The British Journal of Psychiatry, 178(S40), s91–s101. 10.1192/bjp.178.40.s91 11315233

[R5] Copeland WE, Alaie I, Jonsson U, & Shanahan L (2021). Associations of childhood and adolescent depression with adult psychiatric and functional outcomes. Journal of the American Academy of Child & Adolescent Psychiatry, 60(5), 604–611. 10.1016/j.jaac.2020.07.895 32758528 PMC8051642

[R6] Cravens H (1978). The triumph of evolution: The heredity–environment controversy, 1900–1941 (pp. xxiv, 351). Johns Hopkins University Press.

[R7] Cree RA (2018). Health care, family, and community factors associated with mental, behavioral, and developmental disorders and poverty among children aged 2–8 years—United States, 2016. MMWR. Morbidity and Mortality Weekly Report, 67. 10.15585/mmwr.mm6750a1 PMC6342550 30571671

[R8] Dalmaijer E (in prep). Twin studies with unmet assumptions are biased towards genetic heritability. 10.1101/2020.08.27.270801

[R9] Davies NM, Holmes MV, & Davey Smith G (2018). Reading Mendelian randomisation studies: A guide, glossary, and checklist for clinicians. BMJ, k601. 10.1136/bmj.k601 PMC6041728 30002074

[R10] de Zeeuw EL, Hottenga J-J, Ouwens KG, Dolan CV, Ehli EA, Davies GE, Boomsma DI, & van Bergen E (2020). Intergenerational transmission of education and ADHD: Effects of parental genotypes. Behavior Genetics, 50(4), 221–232. 10.1007/s10519-020-09992-w 32026073 PMC7355279

[R11] Eaton WW, Martins SS, Nestadt G, Bienvenu OJ, Clarke D, & Alexandre P (2008). The Burden of mental disorders. Epidemiologic Reviews, 30(1), 1–14. 10.1093/epirev/mxn011 18806255 PMC2683377

[R12] Eaves L (1976). The effect of cultural transmission on continuous variation. Heredity, 37(1), 41–57. 10.1038/hdy.1976.64 1066340

[R13] Eichler EE, Flint J, Gibson G, Kong A, Leal SM, Moore JH, & Nadeau JH (2010). Missing heritability and strategies for finding the underlying causes of complex disease. Nature Reviews Genetics, 11(6), 446–450. 10.1038/nrg2809 PMC2942068 20479774

[R14] Eilertsen EM, Jami ES, McAdams TA, Hannigan LJ, Havdahl AS, Magnus P, Evans DM, & Ystrom E (2021). Direct and indirect effects of maternal, paternal, and offspring genotypes: Trio-GCTA. Behavior Genetics, 51(2), 154–161. 10.1007/s10519-020-10036-6 33387132

[R15] Evans DM, Moen G-H, Hwang L-D, Lawlor DA, & Warrington NM (2019). Elucidating the role of maternal environmental exposures on offspring health and disease using two-sample Mendelian randomization. International Journal of Epidemiology, 48(3), 861–875. 10.1093/ije/dyz019 30815700 PMC6659380

[R16] Friedman NP, Banich MT, & Keller MC (2021). Twin studies to GWAS: There and back again. Trends in Cognitive Sciences. 10.1016/j.tics.2021.06.007 PMC8446317 34312064

[R17] Fromm-Reichmann F (1948). Notes on the development of treatment of schizophrenics by psychoanalytic psychotherapy. Psychiatry, 11(3), 263–273. 10.1080/00332747.1948.11022688 18889231

[R18] Ghandour RM, Sherman LJ, Vladutiu CJ, Ali MM, Lynch SE, Bitsko RH, & Blumberg SJ (2019). Prevalence and treatment of depression, anxiety, and conduct problems in US children. The Journal of Pediatrics, 206, 256–267.e3. 10.1016/j.jpeds.2018.09.021 30322701 PMC6673640

[R19] Grotzinger AD, Rhemtulla M, de Vlaming R, Ritchie SJ, Mallard TT, Hill WD, Ip HF, Marioni RE, McIntosh AM, Deary IJ, Koellinger PD, Harden KP, Nivard MG, & Tucker-Drob EM (2019). Genomic structural equation modelling provides insights into the multivariate genetic architecture of complex traits. Nature Human Behaviour, 3(5), 513–525. 10.1038/s41562-019-0566-x PMC6520146 30962613

[R20] Hart SA, Little C, & van Bergen E (2021). Nurture might be nature: Cautionary tales and proposed solutions. NPJ Science of Learning, 6(1), 1–12. 10.1038/s41539-020-00079-z 33420086 PMC7794571

[R21] Honeycutt H (2019). Nature and nurture as an enduring tension in the history of psychology. Oxford Research Encyclopedia of Psychology, 10.1093/acrefore/9780190236557.013.518

[R22] Horn JM, Loehlin JC, & Willerman L (1979). Intellectual resemblance among adoptive and biological relatives: The Texas Adoption project. Behavior Genetics, 9(3), 177–207. 10.1007/BF01071300 496798

[R23] Hyman SE (2018). The daunting polygenicity of mental illness: Making a new map. Philosophical Transactions of the Royal Society of London. Series B, Biological Sciences, 373(1742), 20170031. 10.1098/rstb.2017.0031 29352030 PMC5790829

[R24] Jaffee SR, Strait LB, & Odgers CL (2012). From correlates to causes: Can quasi-experimental studies and statistical innovations bring us closer to identifying the causes of antisocial behavior? Psychological Bulletin, 138(2), 272–295. 10.1037/a0026020 22023141 PMC3268012

[R25] Jami ES, Hammerschlag AR, Bartels M, & Middeldorp CM (2021). Parental characteristics and offspring mental health and related outcomes: A systematic review of genetically informative literature. Translational Psychiatry, 11(1), 1–38. 10.1038/s41398-021-01300-2 33795643 PMC8016911

[R26] Kanner L (1949). Problems of nosology and psychodynamics of early infantile autism. American Journal of Orthopsychiatry, 19(3), 416–426. 10.1111/j.1939-0025.1949.tb05441.x 18146742

[R27] Keller M, Medland S, Duncan L, Hatemi P, Neale M, Maes H, & Eaves L (2009). Modeling extended twin family data I: Description of the cascade model. Twin Research and Human Genetics, 12(1), 8–18. 10.1375/twin.12.1.8 19210175 PMC4070287

[R28] Keller MC, & Coventry WL (2005). Quantifying and addressing parameter indeterminacy in the classical twin design. Twin Research and Human Genetics, 8(3), 201–213. 10.1375/twin.8.3.201 15989748

[R29] Keller MC, Medland SE, & Duncan LE (2010). Are extended twin family designs worth the trouble? A comparison of the bias, precision, and accuracy of parameters estimated in four twin family models. Behavior Genetics, 40(3), 377–393. 10.1007/s10519-009-9320-x 20013306 PMC3228846

[R30] Kemper KE, Yengo L, Zheng Z, Abdellaoui A, Keller MC, Goddard ME, Wray NR, Yang J, & Visscher PM (2021). Phenotypic covariance across the entire spectrum of relatedness for 86 billion pairs of individuals. Nature Communications, 12(1), 1050. 10.1038/s41467-021-21283-4 PMC7886899 33594080

[R31] Kim Y, Balbona JV, & Keller MC (2021). Bias and precision of parameter estimates from models using polygenic scores to estimate environmental and genetic parental influences. Behavior Genetics, 10.1007/s10519-020-10033-9 PMC8093160 33301082

[R32] Kline R (2016). Principles and practice of structural equation modeling: Fourth edition. Guilford publications.

[R33] Kong A, Thorleifsson G, Frigge ML, Vilhjalmsson BJ, Young AI, Thorgeirsson TE, Benonisdottir S, Oddsson A, Halldorsson BV, Masson G, Gudbjartsson DF, Helgason A, Bjornsdottir G, Thorsteinsdottir U, &Stefansson K (2018). The nature of nurture: Effects of parental genotypes. Science, 359(6374), 424–428. 10.1126/science.aan6877 29371463

[R34] Leverton TJ (2003). Parental psychiatric illness: The implications for children. Current Opinion in Psychiatry, 16(4), 395–402. 10.1097/01.yco.0000079218.36371.78

[R35] Martin AR, Kanai M, Kamatani Y, Okada Y, Neale BM, & Daly MJ (2019). Clinical use of current polygenic risk scores may exacerbate health disparities. Nature Genetics, 51(4), 584–591. 10.1038/s41588-019-0379-x 30926966 PMC6563838

[R36] McAdams TA, Neiderhiser JM, Rijsdijk FV, Narusyte J, Lichtenstein P, & Eley TC (2014). Accounting for genetic and environmental confounds in associations between parent and child characteristics: A systematic review of children-of-twins studies. Psychological Bulletin, 140(4), 1138–1173. 10.1037/a0036416 24749497

[R37] Merikangas KR, Nakamura EF, &Kessler RC (2009). Epidemiology of mental disorders in children and adolescents. Dialogues in Clinical Neuroscience, 11(1), 7–20. 19432384 10.31887/DCNS.2009.11.1/krmerikangas PMC2807642

[R38] Peterson RE, Kuchenbaecker K, Walters RK, Chen C-Y, Popejoy AB, Periyasamy S, Lam M, Iyegbe C, Strawbridge RJ, Brick L, Carey CE, Martin AR, Meyers JL, Su J, Chen J, Edwards AC, Kalungi A, Koen N, Majara L, … Duncan LE (2019). Genome-wide association studies in ancestrally diverse populations: Opportunities, methods, pitfalls, and recommendations. Cell, 179(3), 589–603. 10.1016/j.cell.2019.08.051 31607513 PMC6939869

[R39] Pingault J-B, Rijsdijk F, Schoeler T, Choi SW, Selzam S, Krapohl E, O’Reilly PF, & Dudbridge F (2021). Genetic sensitivity analysis: Adjusting for genetic confounding in epidemiological associations. PLOS Genetics, 17(6), e1009590. 10.1371/journal.pgen.1009590 34115765 PMC8238188

[R40] Plomin R (2019). Blueprint, with a new afterword: How DNA makes us who we are. The MIT Press.

[R41] Posthuma D, & Boomsma DI (2000). A note on the statistical power in extended twin designs. Behavior Genetics, 30(2), 147–158. 10.1023/a:1001959306025 10979605

[R42] Rhee SH, & Waldman ID (2002). Genetic and environmental influences on antisocial behavior: A meta-analysis of twin and adoption studies. Psychological Bulletin, 128(3), 490–529. 10.1037/0033-2909.128.3.490 12002699

[R43] Rutter M, Pickles A, Murray R, & Eaves L (2001). Testing hypotheses on specific environmental causal effects on behavior. Psychological Bulletin, 127(3), 291–324. 10.1037/0033-2909.127.3.291 11393298

[R44] Salam RA, Das JK, & Bhutta ZA (2014). Impact of intrauterine growth restriction on long-term health. Current Opinion in Clinical Nutrition & Metabolic Care, 17(3), 249–254. 10.1097/MCO.0000000000000051 24613859

[R45] Schafer JL, & Graham JW (2002). Missing data: Our view of the state of the art. Psychological Methods, 7(2), 147–177. 10.1037/1082-989X.7.2.147 12090408

[R46] Shih RA, Belmonte PL, & Zandi PP (2004). A review of the evidence from family, twin and adoption studies for a genetic contribution to adult psychiatric disorders. International Review of Psychiatry, 16(4), 260–283. 10.1080/09540260400014401 16194760

[R47] Stein A, Pearson RM, Goodman SH, Rapa E, Rahman A, McCallum M, Howard LM, &Pariante CM (2014). Effects of perinatal mental disorders on the fetus and child. The Lancet, 384(9956), 1800–1819. 10.1016/S0140-6736(14)61277-0 25455250

[R48] Torvik FA, Eilertsen EM, McAdams TA, Gustavson K, Zachrisson HD, Brandlistuen R, Gjerde LC, Havdahl A, Stoltenberg C, Ask H, & Ystrom E (2020). Mechanisms linking parental educational attainment with child ADHD, depression, and academic problems: A study of extended families in The Norwegian Mother, Father and Child Cohort Study. Journal of Child Psychology and Psychiatry, and Allied Disciplines, 61(9), 1009–1018. 10.1111/jcpp.13197 31957030 PMC8607471

[R49] Turkheimer E, & Waldron M (2000). Nonshared environment: A theoretical, methodological, and quantitative review. Psychological Bulletin, 126(1), 78–108. 10.1037/0033-2909.126.1.78 10668351

[R50] Turley P, Meyer MN, Wang N, Cesarini D, Hammonds E, Martin AR, Neale BM, Rehm HL, Wilkins-Haug L, Benjamin DJ, Hyman S, Laibson D, &Visscher PM (2021). Problems with using polygenic scores to select embryos. The New England Journal of Medicine, 385(1), 78–86. 34192436 10.1056/NEJMsr2105065 PMC8387884

[R51] Vogler GP, & Cockerham CC (1985). Multivariate path analysis of familial resemblance. Genetic Epidemiology, 2(1), 35–53. 10.1002/gepi.1370020105 4054591

[R52] Warrington NM, Freathy RM, Neale MC, & Evans DM (2018). Using structural equation modelling to jointly estimate maternal and fetal effects on birthweight in the UK Biobank. International Journal of Epidemiology, 47(4), 1229–1241. 10.1093/ije/dyy015 29447406 PMC6124616

[R53] Wray NR, Lin T, Austin J, McGrath JJ, Hickie IB, Murray GK, & Visscher PM (2021). From basic science to clinical application of polygenic risk scores: A primer. JAMA Psychiatry, 78(1), 101–109. 10.1001/jamapsychiatry.2020.3049 32997097

[R54] Wright S (1934). The method of path coefficients. The Annals of Mathematical Statistics, 5(3), 161–215. JSTOR.

[R55] Yang J, Lee SH, Goddard ME, & Visscher PM (2011). GCTA: A tool for genome-wide complex trait analysis. The American Journal of Human Genetics, 88(1), 76–82. 10.1016/j.ajhg.2010.11.011 21167468 PMC3014363

[R56] Young AI, Frigge ML, Gudbjartsson DF, Thorleifsson G, Bjornsdottir G, Sulem P, Masson G, Thorsteinsdottir U, Stefansson K, & Kong A (2018). Relatedness disequilibrium regression estimates heritability without environmental bias. Nature Genetics, 50(9), 1304–1310. 10.1038/s41588-018-0178-9 30104764 PMC6130754

[R57] Zhang G, Bacelis J, Lengyel C, Teramo K, Hallman M, Helgeland O, Johansson S, Myhre R, Sengpiel V, Njolstad PR, Jacobsson B, &Muglia L (2015). Assessing the Causal relationship of maternal height on birth size and gestational age at birth: A Mendelian randomization analysis. PLOS Medicine, 12(8), e1001865. 10.1371/journal.pmed.1001865 26284790 PMC4540580

